# Improvements in Cycling Time Trial Performance Are Not Sustained Following the Acute Provision of Challenging and Deceptive Feedback

**DOI:** 10.3389/fphys.2016.00399

**Published:** 2016-09-22

**Authors:** Hollie S. Jones, Emily L. Williams, David Marchant, S. Andy Sparks, Craig A. Bridge, Adrian W. Midgley, Lars R. Mc Naughton

**Affiliations:** ^1^School of Psychology, University of Central LancashirePreston, UK; ^2^School of Sport, Leeds Beckett UniversityLeeds, UK; ^3^Department of Sport and Physical Activity, Edge Hill UniversityOrmskirk, UK

**Keywords:** feedback, previous performance, pacing, deception, cycling, endurance performance, self-efficacy

## Abstract

The provision of performance-related feedback during exercise is acknowledged as an influential external cue used to inform pacing decisions. The provision of this feedback in a challenging or deceptive context allows research to explore how feedback can be used to improve performance and influence perceptual responses. However, the effects of deception on both acute and residual responses have yet to be explored, despite potential application for performance enhancement. Therefore, this study investigated the effects of challenging and deceptive feedback on perceptual responses and performance in self-paced cycling time trials (TT) and explored whether changes in performance are sustained in a subsequent TT following the disclosure of the deception. Seventeen trained male cyclists were assigned to either an accurate or deceptive feedback group and performed four 16.1 km cycling TTs; (1 and 2) ride-alone baseline TTs where a fastest baseline (FBL) performance was identified, (3) a TT against a virtual avatar representing 102% of their FBL performance (PACER), and (4) a subsequent ride-alone TT (SUB). The deception group, however, were initially informed that the avatar accurately represented their FBL, but prior to SUB were correctly informed of the nature of the avatar. Affect, self-efficacy and RPE were measured every quartile. Both groups performed PACER faster than FBL and SUB (*p* < 0.05) and experienced lower affect (*p* = 0.016), lower self-efficacy (*p* = 0.011), and higher RPE (*p* < 0.001) in PACER than FBL. No significant differences were found between FBL and SUB for any variable. The presence of the pacer rather than the manipulation of performance beliefs acutely facilitates TT performance and perceptual responses. Revealing that athletes' performance beliefs were falsely negative due to deceptive feedback provision has no effect on subsequent perceptions or performance. A single experiential exposure may not be sufficient to produce meaningful changes in the performance beliefs of trained individuals beyond the acute setting.

## Introduction

During self-paced endurance exercise, athletes will adopt a pacing strategy in the endeavor to produce an optimal performance whilst preventing the occurrence of premature fatigue (Abbiss and Laursen, 2008; Hettinga et al., 2012). Performance-related feedback from external sources is interpreted in accordance with the current pace, internal physiological feedback and the task goals. This integration of information will then govern the continuous and dynamic process of during-task decision-making regarding pace (Renfree et al., 2014; Smits et al., 2014). When external feedback is interpreted in relation to an individual's beliefs in their ability to meet the task demands, it has the potential to elicit positive or negative perceptual experiences (Hutchinson et al., 2008) and performance outcomes (Halson and Martin, 2013). Furthermore, strong beliefs regarding an individual's abilities in their performance have been positively associated with motor performance (McKay et al., 2012), maximal force production (Ness and Patton, 1979; Kalasountas et al., 2007), running efficiency (Stoate et al., 2012), effort tolerance (Hutchinson et al., 2008), and positive affect (McAuley and Courneya, 1992; Stoate et al., 2012).

Previous experience, and the appraisal of the success/failure of this prior effort, will significantly influence an individual's beliefs in their abilities to meet the demands of a similar, future task (Bandura, 1997; Sitzmann and Yeo, 2013). In addition to efficacious beliefs, this prior experience is also considered to be a key determinant of pacing strategies in endurance performance (Micklewright et al., 2010). Therefore it is of interest to explore how prior performance beliefs can be manipulated to enhance self-efficacy and improve future performances. To date, however, few studies have manipulated these beliefs and assessed the residual effects in future exercise bouts. Deception is one method by which these beliefs can be manipulated in order to explore the effects on pacing decisions and performance in self-paced exercise (Jones et al., 2013). For example, the provision of false external feedback prior to or during an exercise bout allows self-beliefs to be surreptitiously augmented in order for behavior to be examined without the influence of unwanted expectancies.

Deceptive conditions present situations of challenge or threat, as described by the Biopsychosocial (BPS) model or, within this exertive context, the Theory of Challenge and Threat States in Athletes (TCTSA) (Blascovich et al., 2004; Jones et al., 2009). These theories propose that threat states occur when an individual perceives that the demands of the task cannot be met by available resources. The nature of deception means that either knowledge of the exercise duration/distance is surreptitiously manipulated (i.e., task demands), or individuals are unknowingly misled as to the level of their own ability (i.e., resources). An example of the latter can be seen in a recent study where deceptive feedback was used to manipulate cyclists' knowledge of their prior time trial performance (Jones et al., 2016). The presence of a visual avatar that unknowingly represented a performance 2% faster than the athletes' baseline effort created a threatening situation for the self. Although performance improved in comparison to the baseline, this improvement was of an equal amount to athletes who knowingly rode against an avatar of their exact baseline performance. This control group, however, may not have experienced a challenge state if the magnitude of the feedback provided did not encourage a motivational focus on success (Blascovich et al., 2004). Challenge states have previously elicited enhanced performances, therefore providing athletes with accurate knowledge of challenging feedback, for example a 2% faster avatar, may reveal the extent to which deception alone may influence performance.

Challenge and threat states have also been shown to influence cognitive processes as well as behavior, where an individual's motivation may be focussed on success in a challenge state and on the prevention of failure in a threat state (Blascovich et al., 2004). These positive and negative motivational states have been found to influence effort, emotions, decision-making, efficacious appraisals and physiological responses (Skinner and Brewer, 2002; Meijen et al., 2013; Vine et al., 2013) and could therefore explain previous findings of altered perceptual responses, such as RPE and affect, in deceptive conditions (Stone et al., 2012; Jones et al., 2016). In the previously mentioned study by Jones et al. (2016), the deception group experienced more negative affect and higher perceived exertion than the control group which supports these aforementioned patterns within threat states. Identifying what factors drive the nature of these perceptual and behavior changes will allow for a greater understanding of the effects of deceptive, or threatening, feedback and its potential application as a training tool.

Most prior deception research has investigated within-trial acute effects of this feedback provision, therefore the residual effects of deceptive interventions are relatively unknown. According to self-efficacy theory (Bandura, 1977), success in a previous performance is thought to most greatly strengthen efficacious perceptions and bring about behavior change (Hutchinson et al., 2008). In instances where deceptive feedback elicits an improvement in performance, unless the athlete can knowingly and accurately appraise their performance as being successful, self-efficacy and resultant behavior changes may not be sustained in future efforts. This is also reverberated by the theory of Psychological Momentum, where expectations of win/loss outcomes are determined as a function of recent successes or failure (Hubbard, 2015). Due to the very nature of deception, individuals may not be explicitly aware that they have performed beyond what they believed possible if the deception remains concealed prior to a future performance. This is supported by the absence of a performance change in a subsequent trial following the provision of deceptive feedback in Jones et al.'s (2016) study, although efficacious experiences were more positive. It is therefore of interest to explore how the disclosure of a deceptive intervention, and the conscious and accurate appraisal of the outcome of a previous performance, may influence self-efficacy and performance in a subsequent performance.

A recent study assessed the residual effects of deception by revealing the true nature of the deception to participants prior to a subsequent trial (Shei et al., 2016). Cyclists were provided with false feedback of a baseline time trial performance using a visual avatar to unknowingly represent 102% of their average baseline power output. Participants then performed a subsequent TT following the disclosure of the deception where, again, they performed against the 102% avatar but this time with accurate knowledge. The resultant performance times were faster in the deception and subsequent TTs compared to the baseline. The authors concluded that the improved performance in the deception trial could be sustained after participants had been informed of the deception. What Shei et al. (2016) did not acknowledge however, is that the mere presence of the avatar in both TTs was likely to have had a motivational influence and could alone explain the faster performances (Williams et al., 2014, 2015; Jones et al., 2016). The lack of a control group and the use of a static avatar set as a depiction of the average baseline power output further limits the study, as indeed it also does in some other deception research (Stone et al., 2012).

The first aim of the present study was to investigate the effects of challenging vs. threatening performance feedback on perceptual responses and performance in 16.1 km self-paced cycling TTs. Secondly, we aimed to explore the residual effects of this acute feedback provision, following the correction of false beliefs incurred via deception. It was hypothesized that (1) performance would be improved with the presence of performance feedback, regardless of feedback accuracy, and (2) the deception group would maintain the performance improvement in a subsequent trial and experience more positive perceptual responses following the disclosure of the deception.

## Methods

### Participants

Seventeen trained male cyclists with race experience in 16.1 km TTs volunteered for the study. Match-paired, random allocation was used to allocate participants to either an accurate (ACC; *N* = 9) or deceptive (DEC; *N* = 8) feedback group based on VO_2peak_ values and performance times attained in TT1 (Table 1). Participants provided prior written informed consent in accordance with the Declaration of Helsinki and the study was approved by Edge Hill University's research ethics committee. Participants were classified as “trained” according to VO_2peak_ and peak power output values (De Pauw et al., 2013).

**Table 1:** **Mean (SD) descriptive data for the ACC and DEC experimental groups**.

	**ACC group (/N= 9)**	**DEC group (/N= 8)**
Age (yrs)	33.0 (6.0)	37.9 (6.5)
Height (cm)	180.0 (3.1)	178.5 (6.7)
Body mass (kg)	77.2 (5.9)	79.4 (5.4)
Absolute PPO (W)	371 (35)	380 (24)
Relative PPO (W/kg)	4.8 (0.5)	4.8 (0.4)
Absolute VO_2peak_ (L·min^−1^)	4.1 (0.4)	4.2 (0.3)
Relative VO_2peak_ (mL·kg·min^−1^)	54.1 (5.9)	53.3 (4.4)

*PPO, peak power output; VO_2peak_, peak oxygen uptake*.

### Research design

**Figure 1:**
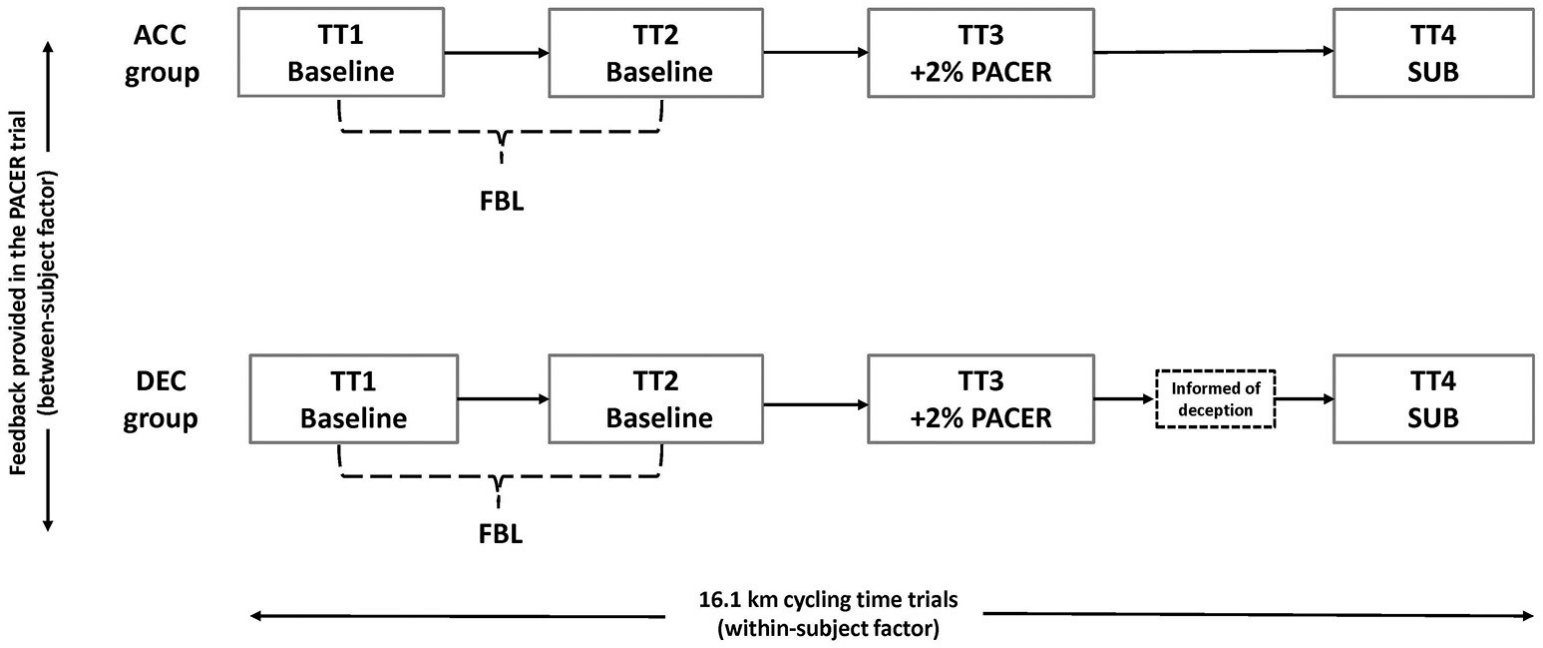
**Trial schematic of the research design**.

A 2 × 3 (group × trial) between- and within-subject experimental design was adopted and participants visited the laboratory on five separate occasions, 2–7 days apart and at the same time of day (±2 h). All visits were completed within a 3 week period and the final trial was completed no more than 7 days after the penultimate visit. A maximal incremental test was completed on the first visit, before both groups completed four 16.1 km cycling TTs (Figure 1).

### Maximal incremental test

Height and body mass were recorded on the participants' initial visit followed by a continuous incremental ramp test to maximal exertion on a cycle ergometer (Excalibur Sport, Lode, Groningen, The Netherlands) to determine VO_2peak_. A 5 min warm-up was performed at 100 W and then initial workloads were determined using established guidelines (Wooles et al., 2003). Increments of 20 W were applied every minute until the required power output could no longer be maintained. Breath-by-breath pulmonary ventilation and gas exchange data were measured throughout the test (Oxycon Pro, Jaeger, GmbH, Hoechburg, Germany) to determine oxygen consumption, which was normalized to pre-exercise body mass data. The VO_2peak_ was defined as the highest VO_2_ value recorded over a 20 s period. Heart rate (Polar Team System, Finland) was recorded continuously using a 5 s sampling rate and verbal encouragement was provided.

### Experimental trials

All participants performed four self-paced 16.1 km TT on their own bicycles, using a calibrated electromagnetically-braked cycle ergometer (CompuTrainer Pro™, RacerMate, Seattle, USA); previously shown to be a reliable measure of power output (Stone et al., 2011). A 0.6% coefficient of variation was found in our laboratory for between-trial variation in performance times (*n* = 31) and a 0.6% smallest worthwhile change in road TT performance has been previously reported (Paton and Hopkins, 2006). The first two TTs (TT1, TT2) were used for familiarization, but to prevent sub-maximal efforts being produced, participants were not informed of this. A flat, virtual course was projected onto a 230 cm screen in front of the rider by the ergometry software, which depicted the participants' speed profile as a synchronized graphical avatar. Time and power output were recorded at a rate of 34 Hz, but distance covered was the only variable displayed. After a 10 min warm-up at 70% of HR_max_, the drafting option in the software was disabled and participants were instructed to complete each TT in the fastest time possible.

**Figure 2:**
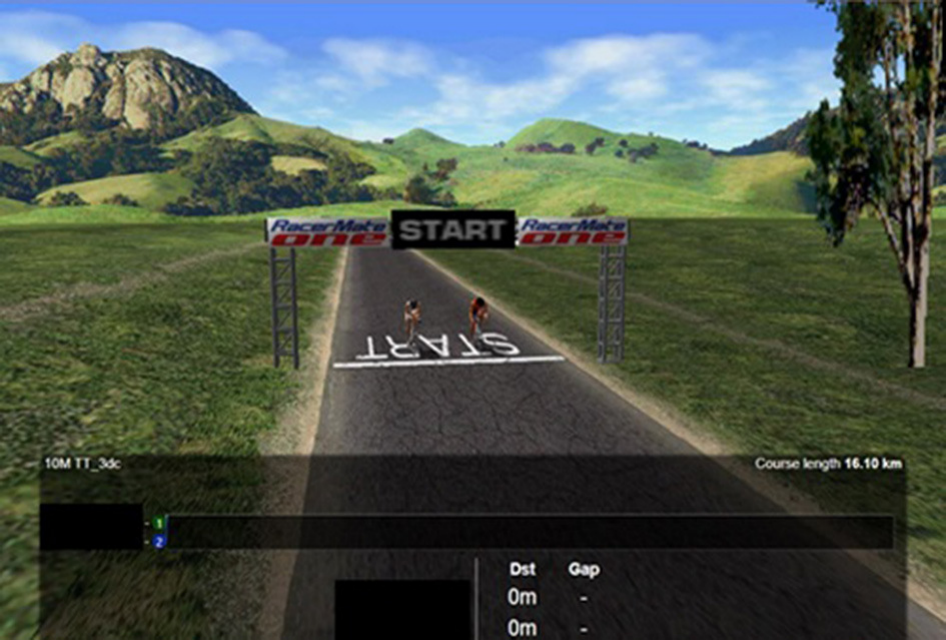
**Representation of the visual feedback provided in the PACER TT**.

Each individual's fastest performance from the two baseline trials was classified as their “fastest baseline” (FBL) and used in all subsequent analysis. In the third TT (PACER), the software represented each participants' FBL performance profile on the screen as a pacer alongside their current performance, depicted as a dynamic and exact replication of the FBL speed profile (Figure 2). In addition to total distance covered, the distance between the participants' avatar and the pacer was also displayed onscreen for both groups. Participants in the ACC group were correctly informed that this pacer was 2% faster than their own FBL performance. In contrast, the pacer in the DEC group also represented a performance corresponding to 2% faster than their FBL but participants were told that it was an accurate representation of their FBL performance. On the final visit, a subsequent TT (SUB) was performed, which was an exact replication of the FBL procedures with no pacer in either group and distance covered feedback only. Immediately before participants in the DEC group commenced their SUB TT, they were informed of the true nature of the pacer that they had performed with in their previous trial. Identical information was given verbally to each participant which stated that the pacer had not represented their fastest baseline TT but had in fact been set 2% faster. No other feedback relating to their performances was provided.

### Perceptual responses

Participants were fully briefed with the instructions for the use of affect, RPE and self-efficacy scales. Affect was measured using the validated 11-point Feeling Scale ranging from +5 (very good) to −5 (very bad) (Hardy and Rejeski, 1989). Participants were informed that their responses should reflect the affective or emotional components of the exercise and not the physical sensations of effort or strain. Borg's (1970) 6–20 scale was used to measure RPE and for task-specific self-efficacy, participants reported “*how confident are you to continue at your current pace for the remaining distance of the trial?”* using a percentage scale from 0% (*cannot do at all*) to 100% (*absolutely certain can do*) (Bandura, 1997; Welch et al., 2010). Verbal responses for affect, RPE and self-efficacy were recorded every 4 km during each TT.

### Physiological variables

Heart rate was measured continuously and respiratory gas analysis recorded expired air every 4 km. Fingertip capillary blood lactate (BLa; Lactate Pro 2, LT-1730, Arkray, Japan) was analyzed prior to each trial and at 4 km intervals.

### Statistical analysis

Linear mixed modeling was used to explore the effects of distance (4, 8, 12, and 16.1 km), trial (FBL, PACER, SUB) and group (ACC, DEC) on all repeated-measures dependent variables; power output, speed, affect, RPE, self-efficacy, heart rate, BLa, V_E_, VO_2_ and RER. Distance, trial and group were modeled as fixed effects and participant as a random effect. Distance was modeled as a continuous variable where linear or quadratic responses were evident, and otherwise modeled as a categorical variable where saturated means modeling was most appropriate. Various plausible covariance structures were assumed, with the structure that minimized the Hurvich and Tsai's criterion (AICC) value chosen for the final fitted model. Performance times were analyzed with fixed effects included for trial and group. Differences between all dependent variables in TT1 and TT2 were analyzed using paired *t*-tests. In the event of significant fixed main or interaction effects, post hoc comparisons with Sidak adjusted *P* values were used to identify significant differences between paired means. Two-tailed statistical significance was accepted as *P* < 0.05 and analyses were conducted using IBM SPSS Statistics 22 (SPSS Inc., Chicago, IL). Descriptive sample statistics are reported as mean and standard deviation (SD) and confidence intervals (CI) are reported at the 95% level. Effect sizes are reported using Cohen's d to indicate the magnitude of the differences between means.

## Results

### Performance variables

Performance times for the ACC group in FBL, PACER and SUB were 26:31 (1:44), 26:15 (1:31), and 26:40 (1:30) min, respectively. For the DEC group, performance times were 26:40 (0:52), 26:22 (0:44), and 26:34 (0:54) min. Performance times between trials were significantly different (*F* = 4.9; *P* = 0.015), with pairwise comparisons indicating that PACER was performed in a significantly faster time than FBL (mean difference (MD) = −17 s; CI = −0.55, −0.01; *P* = 0.042, *d* = 0.20) and SUB (MD = −19 s; CI = −0.59, −0.03; *P* = 0.027, *d* = 0.14; Table 2). Performance time in SUB was not significantly different to FBL (MD = 2 s; CI = −0.24, 0.30; *P* = 0.99, *d* = 0.07). There was not a significant group x trial difference (*F* = 0.7; *P* = 0.49), therefore the differences in performance times between trials were similar in both the ACC and DEC groups.

**Table 2:** **Mean (SD) physiological responses at each distance quartile in 16.1 km time trials for the ACC and DEC groups**.

	**ACC group**				**DEC group**			
	**4 km**	**8 km**	**12 km**	**16.1 km**	**4 km**	**8 km**	**12 km**	**16.1 km**
**HEART RATE (beats·min^−1^)**								
FBL	157 (14)	164 (14)	167 (14)	169 (13)	145 (8)	154 (13)	157 (14)	160 (14)
PACER	160 (9)	169 (10)	170 (11)	172 (10)	147 (9)	158 (12)	160 (13)	162 (13)
SUB	155 (14)	163 (13)	164 (12)	167 (12)	145 (8)	155 (11)	157 (12)	160 (13)
**V_E_ (L·min^−1^)**								
FBL	120.5 (28.3)	121.4 (30.7)	120.0 (31.0)	138.0 (35.4)	127.5 (33.1)	127.2 (33.8)	127.0 (33.9)	151.6 (32.1)
PACER	131.5 (30.9)	132.4 (35.7)	136.7 (38.7)	143.4 (37.4)	136.9 (35.7)	137.8 (30.6)	137.0 (29.0)	154.5 (21.4)
SUB	120.9 (22.9)	117.5 (25.8)	120.2 (31.6)	147.6 (34.0)	125.6 (25.9)	126.8 (19.4)	125.8 (19.8)	147.1 (23.4)
**VO_2_ (L·min^−1^)**								
FBL	3.5 (0.5)	3.5 (0.6)	3.4 (0.6)	3.6 (0.6)	3.6 (0.4)	3.5 (0.5)	3.4 (0.5)	3.7 (0.5)
PACER	3.7 (0.5)	3.6 (0.6)	3.5 (0.6)	3.7 (0.6)	3.7 (0.2)	3.6 (0.4)	3.5 (0.3)	3.7 (0.2)
SUB	3.5 (0.4)	3.4 (0.5)	3.4 (0.5)	3.8 (0.5)	3.5 (0.2)	3.5 (0.3)	3.5 (0.3)	3.7 (0.3)
**RER**								
FBL	1.11 (0.04)	1.12 (0.04)	1.11 (0.04)	1.15 (0.08)	1.12 (0.08)	1.14 (0.08)	1.13 (0.07)	1.19 (0.09)
PACER	1.19 (0.05)	1.15 (0.04)	1.14 (0.03)	1.16 (0.04)	1.20 (0.10)	1.16 (0.10)	1.14 (0.10)	1.18 (0.10)
SUB	1.13 (0.06)	1.09 (0.07)	1.08 (0.07)	1.16 (0.10)	1.21 (0.05)	1.17 (0.04)	1.15 (0.05)	1.20 (0.06)
**BLa (mmol·L^−1^)**								
FBL	7.8 (3.3)	8.9 (3.0)	8.7 (2.9)	9.1 (3.3)	10.5 (3.7)	10.4 (4.4)	11.1 (1.5)	10.8 (5.1)
PACER	8.9 (2.4)	8.9 (3.3)	9.4 (3.9)	9.7 (3.5)	11.4 (4.6)	12.1 (5.1)	12.3 (5.3)	12.2 (4.1)
SUB	6.7 (2.6)	6.1 (3.3)	6.6 (4.2)	9.3 (4.6)	10.7 (4.6)	10.7 (4.9)	10.4 (4.8)	11.0 (4.2)

*V_E_, minute ventilation; VO_2_, pulmonary oxygen uptake; RER, respiratory exchange rate; BLa, blood lactate*.

**Figure 3:**
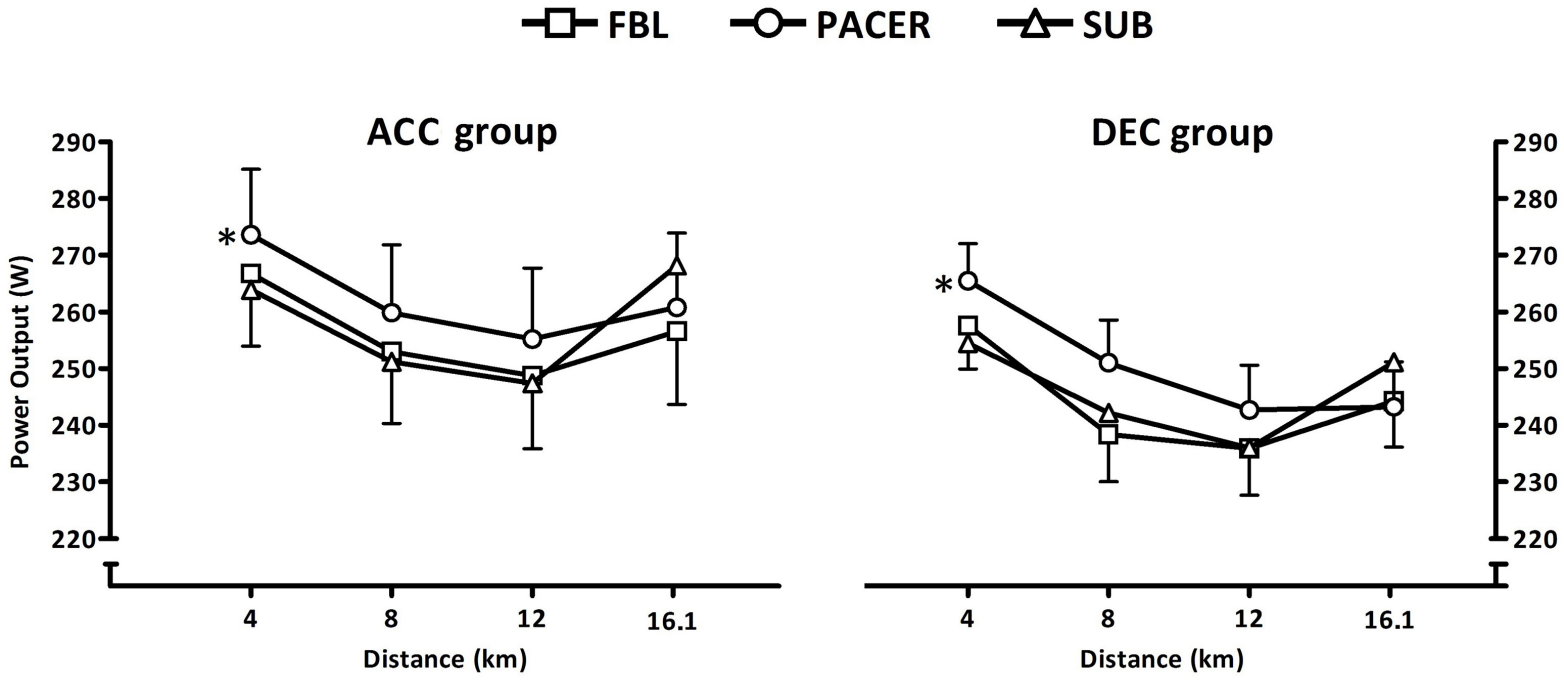
**Mean (SEM) power output at each distance quartile in 16.1 km time trials for the ACC and DEC groups**. ^*^Denotes significantly higher mean power output than FBL and SUB (*P* < 0.001).

Significant main effects for power output were found for distance (*F* = 91.9; *P* < 0.001) and trial (*F* = 9.2; *P* < 0.001). Post hoc analysis revealed that PACER was performed at a significantly higher power output than both FBL (MD = 7 W; CI = 3.17, 10.70; *P* < 0.001; *d* = 0.22) and SUB (MD = 8 W; CI = 4.34, 12.03; *P* < 0.001; *d* = 0.27). Similarly, main effects for speed were found for distance (*F* = 29.9; *P* < 0.001) and trial (Speed: *F* = 7.0; *P* = 0.001). Speed was significantly higher in PACER than both FBL (MD = 0.4 km·hr^−1^; CI = 0.16, 0.59; *P* < 0.001; *d* = 0.20) and SUB (MD = 0.4 km·hr^−1^; CI = 0.19, 0.64; *P* < 0.001; *d* = 0.13). No significant group × trial interactions were found for power output or speed (PO: *F* = 0.4; *P* = 0.69, Speed: *F* = 0.3; *P* = 0.72) indicating that pacing strategies in each trial were similar between the ACC and DEC groups (Figure 3).

### Perceptual responses

**Figure 4:**
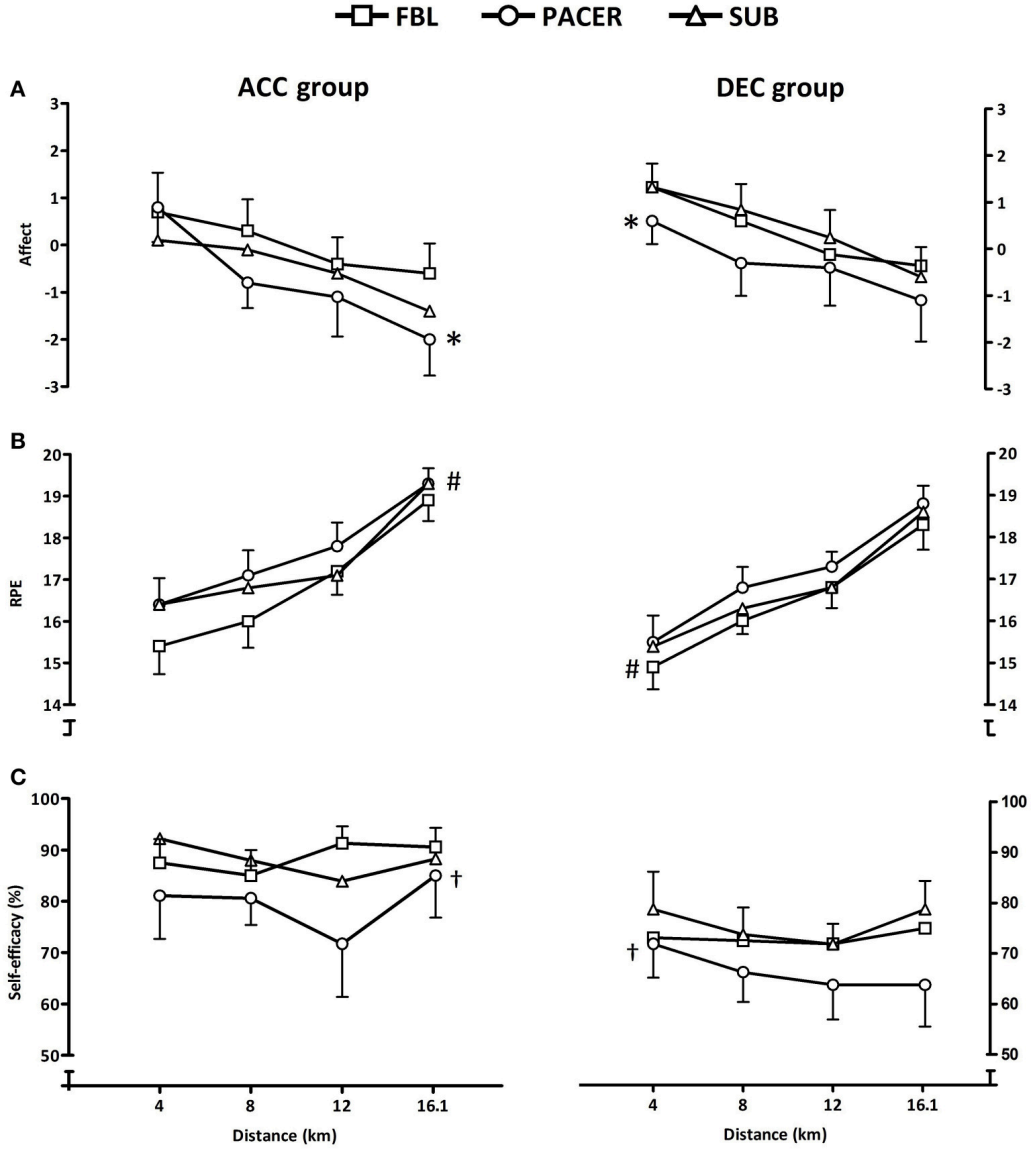
**Mean (SEM) affect (A), RPE (B), and self-efficacy (C) responses at each distance quartile in 16.1 km time trials for the ACC and DEC groups**. ^*^Denotes significantly lower mean affect than FBL (*P* < 0.005). ^#^Denotes significantly higher mean RPE than FBL and SUB (*P* < 0.005). ^†^Denotes significantly lower mean self-efficacy than FBL and SUB (*P* < 0.005).

Significant main effects for affect were found for distance (*F* = 16.3; *P* < 0.001) and trial (*F* = 4.5; *P* = 0.02), with significantly lower affect in PACER than FBL (MD = −0.69; CI = −1.28, −0.11; *P* = 0.016, *d* = 0.95; Figure 4A). Main effects for RPE were similarly found for distance *F* = 14.2; *P* < 0.001) and trial (*F* = 4.6; *P* = 0.012). RPE in PACER was significantly higher than in FBL (MD = 0.7; CI = 0.34, 1.04; *P* < 0.001, *d* = 0.36) and SUB (MD = 0.4, CI = 0.07, 0.78; *P* = 0.014, *d* = 0.23; Figure 4B). For self-efficacy, significant group (*F* = 4.9; *P* = 0.042) and trial (*F* = 8.9; *P* = 0.001) main effects were found, showing that the DEC group were significantly less confident than the ACC group (MD = −14.2%; CI = −27.81, -0.55; *P* = 0.042, *d* = 2.61). Self-efficacy was lower in PACER than FBL (MD = −7.6%; CI = −13.76, −1.48; *P* = 0.011, *d* = 0.97) and SUB (MD = −10.0%; CI = −16.13, −3.82; *P* = 0.001, *d* = 1.22; Figure 4C).

### Physiological variables

Significant main effects for heart rate were found for trial (*F* = 7.5; *P* = 0.002) and distance (*F* = 57.7; *P* < 0.001). Significantly higher values were found in PACER than FBL (MD = 3 beats·min^−1^; CI = 0.51, 6.44; P = 0.017, *d* = 0.25) and SUB (MD = 4 beats·min^−1^; CI = 1.42, 7.45; *P* = 0.002, *d* = 0.36). A significant trial x distance interaction was also found (*F* = 2.7; *P* = 0.036) and post hoc analysis revealed significantly higher heart rate in PACER than FBL at 8 km (MD = 5 beats·min^−1^; CI = 0.21, 0.57; *P* = 0.021, *0* = 0.35) and in PACER than SUB at 8 km (MD = 5 beats·min^−1^; CI = 0.51, 8.97; *P* = 0.024, *d* = 0.42) and 12 km (MD = 5 beats·min^−1^; CI = 1.03, 9.51; *P* = 0.01, *d* = 0.44). Significant main effects for V_E_ were found for distance (*F* = 20.20,*P* < 0.001) and trial (*F* = 9.6; *P* < 0.001). V_E_ in PACER was significantly higher than in FBL (MD = 9.6 L·min^−1^; 95 = 1.74, 17.50; *P* = 0.012, *d* = 1.13) and SUB (MD = 13.6 L·min^−1^; 95 = 5.37, 21.78; *P* < 0.001, *d* = 0.29). A significant main effect for distance was found for RER (*F* = 56.5, *P* < 0.001) and both trial (*F* = 4.0; *P* < 0.029) and distance (*F* = 21.2, *P* < 0.001) main effects were found for VO_2_. Mean VO_2_ was significantly higher in PACER than SUB (MD = 12.6 L·min^−1^; CI = 7.77, 243.82; *P* = 0.033, *d* = 1.13) (Table 2). A significant main effect for BLA was found for trial (*F* = 6.3; *P* = 0.005), with higher values found in PACER than SUB (MD = 1.6 mmol·L^−1^; CI = 0.46, 2.72; *P* = 0.003, *d* = 0.23). The difference in BLa between PACER and FBL was also approaching significance (MD = 1.1 mmol·L^−1^; CI = −0.04, 2.19; *P* = 0.062, *d* = 0.83; Table 2).

### TT1–TT2

Between-group analysis for TT1 and TT2 data revealed no significant differences for performance time, RPE, self-efficacy, V_E_, VO_2_, RER or BLa (*P* > 0.083). In the ACC group, power output and speed were significantly higher at 4 km in TT1 than TT2 (PO: MD = 9 W; CI = 1.2, 18.2; *P* = 0.03, Speed: MD = 0.5 km.h-1; CI = 0.03, 0.92; *P* = 0.038). Heart rate was significantly higher in TT1 than TT2 for both groups. In the ACC group, heart rate was higher at 4 and 8 km (*P* < 0.008), and at 4, 8 and 12 km in the DEC group (*P* < 0.029). A significant difference was found in the DEC group for affect at 16.1 km, with a higher value found in TT2 than TT1 (MD = 1.3; CI = 0.18, 2.32; *P* = 0.028). Nine participants performed TT1 faster than TT2 and eight participants performed TT2 in a faster time.

## Discussion

The main findings demonstrate that cycling TT performance is not influenced by the manipulation of previous performance beliefs and is instead facilitated acutely via the provision of visual feedback. Both the ACC and DEC groups equally improved performance with the presence of a pacer representative of a 2% faster performance than their FBL. Similarly, both groups experienced lower affect and self-efficacy and higher RPE in this PACER trial. The novel inclusion of a subsequent trial following the disclosure of the nature of the deception aimed to evaluate whether the manipulation of beliefs can elicit an enduring change of behavior and perceptual appraisals. However, residual effects of this feedback provision were absent in both groups as neither perceptions nor performance differed between FBL and SUB. This suggests that the facilitation of a visual avatar has only acute effects which are not sustained residually. This is evident irrespective of whether the avatar is an accurate representation of a 2% faster profile of an athlete's previous performance or whether the athlete falsely believes that this 2% faster avatar represents their previous performance. Even with corrected knowledge of the prior deception, the DEC group's perceptual responses nor performance differed in their SUB TT.

The current study supported previous findings which evidenced acute facilitative effects of visual feedback provision on performance during self-paced cycling TTs (Corbett et al., 2012; Stone et al., 2012; Williams et al., 2014, 2015; Jones et al., 2016). Both groups performed against the same magnitude of pacer (102% of FBL) but were provided with different instructions and therefore had different pre-performance beliefs. The key findings indicate that the presence of a pacer improves performance but the accuracy of the feedback provided, and thus the participants' beliefs, had no effect on the extent of this improvement. This is in support of a recent study which also demonstrated that TT performance improvements were similar between deception and control groups despite differences in performance beliefs (Jones et al., 2016). Furthermore, whilst supporting the first hypothesis, physiological and perceptual responses did not differ between groups; RPE, heart rate, BLa, V_E_ and VO_2_ all increased in PACER and affect and self-efficacy were lower, further indicating that beliefs did not influence other variables. The ACC group were able to focus on success without the threat of failure as they had accurate knowledge of the augmented performance feedback and, therefore, a more socially acceptable failure outcome. Contrastingly, the DEC group were exposed to threat as they were misinformed about the augmented feedback. In this case, the failure to match a performance believed to be achievable would not be considered an acceptable outcome by these trained, competitive cyclists. Interestingly, this threat state did not result in a slower performance or more negative perceptual responses, as supported by previous evidence of differences in perceptual and behavior outcomes between challenge and threat situations (Skinner and Brewer, 2002; Meijen et al., 2013; Vine et al., 2013). Consequently, activating motivational processes via the use of challenging/threatening visual feedback appears to be more effective at improving performance than conditions of no activation. However, the neurological mechanisms of these motivational processes might differ and warrant exploration in future research.

The absence of a difference between groups is in contrast to previous research in which deceptive exposures have elicited performance improvements beyond that of a control condition (Corbett et al., 2012; Stone et al., 2012). Stone et al. (2012) highlighted the potentially confounding effect of social facilitation on the findings and acknowledged that an accurately informed group competing against a 102% pacer would reveal the extent to which competition alone may have influenced their findings. The inclusion of a 102% accurate feedback group in this study, and resultant findings that TT performance did not differ to the DEC group, therefore supports that simply the presence of challenging visual feedback is sufficient to evoke a faster TT performance, and not the beliefs associated with the feedback (Weinberg et al., 1979, 1981).

The true nature of the deception was revealed to the participants in the DEC group prior to completion of the SUB TT. This information acted to correct the false belief that they had performed worse in PACER in comparison to their FBL. Similarly, however, performance and perceptions following this disclosure did not vary in comparison to the ACC group, refuting our second hypothesis. This differs from the findings of Shei et al. (2016) who used a feedback manipulation which was also revealed to participants and observed performance improvements. Instead, our data suggest that these results were likely confounded by the presence of the pacer in the subsequent trial. In the present study, the absence of between-group differences did not demonstrate that the correction of false beliefs, intended to produce positive beliefs and stimulate psychological momentum, influenced cycling TT performance or perceptual responses. This may be explained by the explication of the deception disclosure. Participants were simply informed that the feedback provided in PACER was false but no explicit reference was made to the performance outcome, such as completion time. Therefore, it cannot be concluded that all participants reappraised their prior performance as successful and thus inducing a positive effect on self-efficacy.

In summary, the practical implications of feedback provision, either accurate and challenging or non-contingent and threatening in nature, may be subject to the success or failure of the performance during the exposure and thus is an area warranting further exploration. It should be noted that a true control condition was not included in this study, i.e., accurate feedback of a pacer representing the participants' FBL performance, therefore the reader should also consider previous findings in their evaluation of the current study (see Stone et al., 2012; Jones et al., 2016). Finally, it is possible that a single experiential exposure may not be sufficient to produce meaningful changes in the performance beliefs of trained individuals beyond the acute setting and future research should explore the manipulation of beliefs over longer periods.

## Conclusion

The main findings from this study extend support that deception has no additional influence on 16.1 km cycling TT performance or perceptual responses than simply the presence of challenging feedback. This therefore suggests that the accuracy of visual feedback provided to athletes and the resultant performance beliefs might be superfluous. Revealing to athletes that their prior performance beliefs were falsely negative due to an exposure to deceptive feedback has no effect on subsequent perceptions or performance.

## Author contributions

HJ, Conceptualizing and designing the study, data acquisition, data analysis, drafting the article, revising it critically for intellectual content, final approval of the version to be published, agreement to be accountable for all aspects of the work; EW, Conceptualizing and designing the study, data acquisition, final approval of the version to be published; DM, revising it critically for intellectual content, final approval of the version to be published, agreement to be accountable for all aspects of the work; SS, Interpretation of the data, revising it critically for intellectual content, final approval of the version to be published, agreement to be accountable for all aspects of the work; CB, Interpretation of the data, revising it critically for intellectual content, final approval of the version to be published, agreement to be accountable for all aspects of the work; AM, Data analysis, revising it critically for intellectual content, final approval of the version to be published, agreement to be accountable for all aspects of the work; LM, Interpretation of the data, revising it critically for intellectual content, final approval of the version to be published, agreement to be accountable for all aspects of the work.

### Conflict of interest statement

The authors declare that the research was conducted in the absence of any commercial or financial relationships that could be construed as a potential conflict of interest.
